# Association of *IL33*, *IL1RL1*, *IL1RAP* Polymorphisms and Asthma in Chinese Han Children

**DOI:** 10.3389/fcell.2021.759542

**Published:** 2021-12-15

**Authors:** Maolan Wu, Xiangrong Zheng, Juan Huang, Xiaolei Hu

**Affiliations:** ^1^ Department of Pediatrics, Xiangya Hospital, Central South University, Changsha, China; ^2^ Department of Pediatrics, The First Hospital of Changsha, Changsha, China; ^3^ National Institution of Drug Clinical Trial, Xiangya Hospital, Central South University, Changsha, China

**Keywords:** IL33, IL1RL1, IL1RAP, SNPs, childhood asthma, eosinophilic airway inflammation, ICS, Chinese Han nationality

## Abstract

**Background:** Genome-wide association studies have identified interleukin 33 (*IL33*), interleukin 1 receptor-like 1 (*IL1RL1*), interleukin 1 receptor accessory protein (*IL1RAP*) as asthma susceptibility loci in Europeans. IL33, IL1RL1, and IL1RAP constitute a ligand-receptor complex.

**Objective:** We analyzed associations of asthma susceptibility, eosinophilic airway inflammation, and response to inhaled corticosteroid (ICS) with single nucleotide polymorphisms (SNPs) of 3 genes encoding IL33, IL1RL1, and its coreceptor IL1RAP in Chinese Han nationality children.

**Methods:** A total of 153 non-asthmatic children and 265 asthmatic children who visited the Xiangya Hospital between September 2015 and August 2019 were recruited for this study. Pulmonary function tests, peripheral blood eosinophil counts (PBEC), and fractional exhaled nitric oxide (FeNO) tests were performed before treatment, and 3 months after treatment. Each participant’s DNA was extracted from the peripheral blood, and a Mass ARRAY system was used to genotype the SNPs.

**Results:** The T allele of rs4742170 in *IL33* was associated with a risk of higher FeNO at baseline, and no improvement in FeNO and airway hyperresponsiveness was found after ICS treatment. The A allele of rs10208293 and C allele of rs13424006 in *IL1RL1* both were associated with lower susceptibility to asthma and lower FeNO. The TT genotype of rs1420101 and AA genotype of rs4142132 in *IL1RL1* were associated with a greater probability of improvement in PBEC after ICS treatment.

**Conclusion:** IL33-IL1RL1-IL1RAP complex polymorphisms are associated with childhood asthma susceptibility, eosinophilic airway inflammation, and ICS response in Chinese Han children in Hunan. We speculate that IL33-IL1RL1-IL1RAP complex polymorphisms affect the development of asthma, airway inflammation, and subsequent ICS response in childhood.

## Introduction

Asthma is a heterogeneous disease characterized by chronic airway inflammation and is the most common chronic disease in childhood (El-Husseini et al., 2020; di Palmo et al., 2021). The development of asthma results from a complex interaction between genetic predisposition and environmental triggers (Papi et al., 2018; Wang et al., 2020). In the complex pathogenesis of asthma, estimates of heritability range from 40 to 85%, and are higher for childhood-onset asthma than it is for adult asthma (Morales and Duffy, 2019). Given the extensive heterogeneity of asthma pathogenesis, a different strategy, starting from specific phenotype observations that are more likely to be driven by genetic mechanisms, might help to increase the power of genetic studies (di Palmo et al., 2021).

It is believed that typical eosinophilic airway inflammation, formed through a series of unclear pathophysiological mechanisms, is the dominant inflammation in most asthmatic children and 50% of asthmatic adults (2020). Eosinophilic airway inflammation is a manifestation of type 2 inflammation. IL33 is known as an alarmin cytokine from the IL-1 family of cytokines and has significant roles in type 2 immunity, and diseases such as allergic diseases and refractory asthma (Cayrol and Girard, 2018, 33; Chan et al., 2019, 33; Murdaca et al., 2019, 33; Zheng et al., 2020). It is mainly secreted by the cells of barrier tissues, such as endothelial cells, fibroblasts, and epithelial cells of the skin, gastrointestinal tract, and lungs that are exposed to the environment (Cayrol and Girard, 2018, 33; Chan et al., 2019, 33; Gabryelska et al., 2019). IL33 is an important immunomodulator and is released as an alarm signal upon cell necrosis (Cayrol and Girard, 2018, 33). Major targets of IL33 are mast cells, group 2 innate lymphoid cells (ILC2s), and tissue regulatory T cells (Tregs), that constitutively express membrane receptor ST2, which is encoded by the IIL1RL1 gene (Molofsky et al., 2015; Liew et al., 2016, 33). It has been shown in many studies that IL33 is a critical regulator of these tissue-resident immune cells. IL33 binds to its receptor, ST2, to interact with the IL-1 receptor accessory protein (IL1RAP), a co-receptor made by a central five-stranded sheet surrounded by five helices placed on the cytosolic end of the protein (Martin, 2013, 33). The IL33/ST2/IL1RAP complex then induces target cells, such as mast cells and ILC2s, to release type 2 inflammatory cytokines, promote the differentiation of B cells into IgE-producing cells, and induce eosinophil proliferation, thus forming an internal environment for a type 2 inflammatory reaction in the lung or other tissues (Gasiuniene et al., 2019; Johansson and McSorley, 2019).

Clinically, type 2 airway inflammation is usually characterized by eosinophil increase or fractional exhaled nitric oxide (FeNO) elevation, while type 1 inflammation is usually characterized by the neutrophil increase (2020). ICS is the first-line medicine for long-term control of asthma. Generally, type 2 airway inflammation, which is classified as mild or moderate asthma inflammation, is rapidly improved after regular and correct use of (GINA, 2020). However, the efficacy of ICS has obvious individual differences. Patients who respond poorly to ICS treatment or who are particularly sensitive to ICS, account for a significant proportion of the total population. The wide variability of response to ICS treatment, and the extensive similarity of response among relatives, suggest a genetic basis for the differences in ICS efficacy (Chung et al., 2014). Non-response to ICS treatment is often associated with loss of work, increased family and socioeconomic burden, and so on. The number of patients with type 2 airway inflammation could be as high as 50% of patients with a poor response to ICS. The emerging immune biological agents targeting type 2 provide great hope for the control of ICS-unresponsive asthma (Papi et al., 2018; Chan et al., 2019, 33).

Even though large-scale, genome-wide association studies have demonstrated that the IL33, IL1RL1, and IL1RAP genes are associated with a susceptibility to asthma in Europeans (Savenije et al., 2014; El-Husseini et al., 2020), there is still a lack of studies into the association of gene polymorphisms of the IL33/ST2/IL1RAP complex for other population groups. Therefore, we analyzed the association of asthma susceptibility, eosinophilic airway inflammation, and response to inhaled corticosteroid (ICS), with single nucleotide polymorphisms (SNPs) of IL33, IL1RL1, and IL1RAP in Chinese Han children, to investigate the effects of those three gene polymorphisms on the risk of childhood asthma, types of airway inflammation, and ICS response in asthmatic Chinese children.

## Methods

### Study Population and Design

This case-control study was done in Hunan province, China. Two hundred sixty-five asthmatic children aged 3–14 years and 153 controls were recruited between September 2015 to August 2019 at Xiangya Hospital, Central South University. Diagnosis and treatment are based on Chinese childhood asthma guidelines 2016 (Association Editorial Board Chinese Journal of Pediatrics., 2016), which closely follows the Global Initiative for Asthma (GINA, 2015) (2015). Children with a long history of exposure to second-hand smoke, respiratory infections, and systemic infections within 1 month, diagnosed with congenital lung malformations, airway obstruction, extraluminal oppression, congenital heart disease, active tuberculosis, bronchiectasis, or severe systemic diseases were excluded. In addition to not having an asthma diagnosis, children in the control group did not have a diagnosis of bronchial asthma, bronchiolitis, allergic diseases, severe systemic diseases, or a family history of allergic diseases. Basic demographic information was collected at the inception of the study.

### Measurement of PBEC, FeNO, and Pulmonary Function

PBEC, FeNO, and pulmonary function were measured at the first visit, and again after 3 months of ICS treatment. A PBEC higher than 300 /μL is considered to be elevated (2020). For asthmatic children over 6 years old, Pulmonary function was performed using the Jaeger Masterscope spirometry system (Jaeger, Wuerzburg, Germany). A provocative dose of methacholine causing a 20% drop in FEV1 (PD20) was used to represent airway hyperresponsiveness. Forced expiratory volume in 1s/Forced vital capacity (FEV1/FVC), and percentage of the predicted value of maximal mid-expiratory flow (MMEF/pre), were used to evaluate pulmonary function. A Nakoulon breath machine (Sunvou, China) was used to measure FeNO. A FeNO measurement greater than 25ppb is considered to be elevated (Pulmonary Function Cooperation Group of Respiratory Group Pediatrics Society of Chinese Medical Association Editorial Board of the Chinese Journal of Clinical Applied Pediatrics, 2017).

### SNP Selection and Genotyping

In this study, 10 SNPs in three genes (rs4742170, rs2381416, rs928413, and rs992969 in IL33; rs10208293, rs13424006, rs1420101 and rs4142132 in IL1RL1; rs9290936 and rs10513854 in IL1RAP) were chosen for genotyping, based on their potential functionality and their reported association with asthma (Savenije et al., 2014; Morales and Duffy, 2019; Schoettler et al., 2019). SNPs were excluded if the minor allele frequency in CHB was less than 0.1, or the r^2^ value was less than 0.8.

Genotyping was performed using the iPLEX Mass ARRAY genotyping platform (Sequenom, Inc., San Diego, CA). DNA was extracted from 2 ml of the collected blood using a DNA extraction kit (SQ Blood DNA KitII, Omega, United States). The primers were designed by AssayDesigner4.0.

### Statistical Analysis

SPSS24.0 (SPSS Inc., Tokyo, Japan) was used for statistical analysis. In the analysis of the difference between the genotype frequency and the expected genotype frequency, *p* > 0.05 was considered to be consistent with the Hardy-Weinberg equilibrium (HWE). T-test, Chi-square test, and logistic regression were used to calculate the significance of differences, *p* < 0.05 was considered to be statistically significant.

## Results

### Population Characteristics

From September 2015 to August 2019, 265 asthmatic children (185 males, 80 females, mean age 7.62 ± 2.93 years) and 153 healthy controls (95 males, 58 females, mean age 7.17 ± 2.66 years) were recruited in the outpatient or emergency department of pediatrics and the pediatric ward of Xiangya Hospital, Central South University. There was no significant difference in sex, age, permanent residence, and birth history between the two groups. The detailed baseline demographics of subjects are listed in Table 1.

**TABLE 1:** of children with (case) and without (control) doctor-diagnosed asthma (*n* = 418).

Characteristics	Case(*n* = 265)		Control (*n* = 153)		*p*-value
	N	(%)	N	(%)	
Sex					0.106
Boys	185	(69.81)	95	(62.09)	
Girls	80	(30.19)	58	(37.91)	
Age (years)					0.467
<6	60	(22.64)	30	(19.61)	
≥6	205	(77.36)	123	(80.39)	
Residence					0.271
Rural	73	(27.55)	49	(32.03)	
Urban	192	(72.45)	104	(67.97)	
Gestational age (weeks)					0.235
<37	16	(6.04)	11	(7.19)	
≥37	249	(93.96)	142	(92.81)	
Mode of delivery					0.336
Natural labor	166	(62.64)	103	(67.32)	
Caesarean birth	99	(37.36)	50	(32.68)	
Atopy					<**0.001**
Yes	90	(33.96)	0	(0.00)	
No	175	(66.04)	153	(100.00)	
Parental atopy					<**0.001**
Yes	84	(31.70)	0	(0.00)	
No	181	(68.30)	153	(100.00)	

The values *p* ≤ 0.05 are in bold.

Of the265 children with asthma, 200 received standardized treatment for 3 months, 117 children were selected for examination and re-examination, 103 children were over 6 years old. Table 2 presents a comparison of indicators from before and after treatment. The mean values of PBEC and FeNO decreased after treatment, while the mean values of FEV1/FVC and MMEF/PRE, increased.

**TABLE 2:** Indicators at the first visit and after 3 months of ICS treatment.

Indicator	Baseline	After ICS treatment	*p*-value
PBEC (/μL)	463.64 ± 369.69	382.64 ± 290.12	**0.002**
FeNO(bbp)	46.91 ± 30.66	20.19 ± 15.64	<**0.001**
FEV1/FVC(%)	93.06 ± 11.90	99.13 ± 16.83	**0.002**
MMEF/pre (%)	63.60 ± 24.12	76.24 ± 22.16	<**0.001**
PD20	0.81 ± 0.78	1.17 ± 0.88	**0.035**

ICS: inhaled corticosteroid; PBEC: peripheral blood eosinophil count; FeNO: fractional exhaled nitric oxide; FEV1: forced expiratory volume in 1 s; FVC: forced vital capacity; MMEF: maximal mid-expiratory flow; PD20: provocative dose of methacholine causing a 20% drop in FEV1.*p*-values < 0.05 are in bold.

### Association of IL33-IL1RL1-IL1RAP Complex SNPs With Childhood Asthma Susceptibility

All the SNPs involved in our study were consistent with HWE. The allele and genotype frequencies of the 10 SNPs in asthmatics and controls are listed in Table 3. Table 4 shows the significant results for the susceptibility of children to asthma. We observed that rs10208293 and rs13424006 in IL1RL1 were significantly different between asthmatic children and controls in allele frequencies (*p* = 0.015 and *p* = 0.017, respectively; Table 3). Children with an AA or AG genotype of rs10208293 had a decreased risk for asthma, compared with the other genotypes (corrected OR = 0.577, 95%CI: 0.359-0.927, *p* = 0.023). Children with a CT or CC genotype of rs13424006 (corrected OR = 0.584, 95%CI: 0.362-0.941, *p* = 0.027).

**TABLE 3:** The allele and genotype frequency of 10 SNPs in children with (case) and without (control) doctor-diagnosed asthma.

Gene	SNP	Genotype/Allele	Case (N = 265)		Control (N = 153)		*p*-value	^a^DOM model *p*-value	^b^REC model *p*-value
			N	(%)	N	(%)			
IL33	rs4742170	Total	262	(100.00)	146	(100.00)	0.271	0.174	0.207
		CC	48	(18.32)	35	(23.97)			
		TC	134	(51.15)	75	(51.37)			
		TT	80	(30.53)	36	(24.66)			
		C	230	(43.89)	145	(49.66)	0.113		
		T	294	(56.11)	147	(50.34)			
	rs2381416	Total	261	(100.00)	150	(100.00)	0.812	0.535	0.666
		AA	235	(90.04)	137	(91.33)			
		CA	24	(9.20)	13	(8.67)			
		CC	2	(0.77)	0	(0.00)			
		A	494	(94.64)	287	(95.67)	0.513		
		C	28	(5.36)	13	(4.33)			
	rs928413	Total	262	(100.00)	151	(100.00)	0.504	0.302	0.460
		AA	224	(85.5)	133	(88.08)			
		GA	35	(13.36)	18	(11.92)			
		GG	3	(1.15)	0	(0.00)			
		A	483	(92.18)	284	(94.04)	0.316		
		G	41	(7.82)	18	(5.96)			
	rs992969	Total	264	(100.00)	152	(100.00)	0.555	0.557	0.512
		AA	3	(1.14)	0	(0.00)			
		AG	21	(7.95)	11	(7.24)			
		GG	240	(90.91)	141	(92.76)			
		A	27	(5.11)	11	(3.62)	0.320		
		G	501	(94.89)	293	(96.38)			
IL1RL1	rs10208293	Total	263	(100.00)	152	(100.00)	**0.015**	**0.010**	1.000
		AA	1	(0.38)	1	(0.66)			
		AG	48	(18.25)	44	(28.95)			
		GG	214	(81.37)	107	(70.39)			
		A	50	(9.51)	46	(15.13)	**0.015**		
		G	476	(90.49)	258	(84.87)			
	rs13424006	Total	263	(100.00)	150	(100.00)	**0.025**	**0.012**	1.000
		CC	1	(0.38)	1	(0.67)			
		CT	48	(18.25)	43	(28.67)			
		TT	214	(81.37)	106	(70.67)			
		C	50	(9.51)	45	(15.00)	**0.017**		
		T	476	(90.49)	255	(85.00)			
	rs1420101	Total	262	(100.00)	149	(100.00)	0.396	0.605	0.303
		CC	82	(31.30)	43	(28.86)			
		TC	127	(48.47)	82	(55.03)			
		TT	53	(20.23)	24	(16.11)			
		C	291	(55.53)	168	(56.38)	0.815		
		T	233	(44.47)	130	(43.62)			
	rs4142132	Total	262	(100.00)	148	(100.00)	0.482	0.416	0.266
		AA	63	(24.05)	43	(29.05)			
		GA	133	(50.76)	73	(49.32)			
		GG	66	(25.19)	32	(21.62)			
		A	259	(49.43)	159	(53.72)	0.238		
		G	265	(50.57)	137	(46.28)			
IL1RAP	rs9290936	Total	263	(100.00)	150	(100.00)	0.887	0.637	0.994
		GG	83	(31.56)	44	(29.33)			
		GT	138	(52.47)	82	(54.67)			
		TT	42	(15.97)	24	(16.00)			
		G	304	(57.79)	170	(56.67)	0.753		
		T	222	(42.21)	130	(43.33)			
	rs10513854	Total	254	(100.00)	130	(100.00)	0.742	1.000	0.537
		TT	5	(1.97)	3	(2.31)			
		TC	65	(25.59)	29	(22.31)			
		CC	184	(72.44)	98	(75.38)			
		T	75	(14.76)	35	(13.46)	0.626		
		C	433	(85.24)	225	(86.54)			

If the total number of cases is not 265 or the number of controls is not 135, it is due to missing data. *p*-values < 0.05 are in bold.

a DOM, means AA vs (Aa + aa).

b REC, means (AA+ Aa) vs aa; “A” is the major allele and “a” is the minor allele.

**TABLE 4:** Association of gene SNPs with asthma of children susceptibility.

SNP	OR (95%CI)	OR corr^a^	
rs4742170 DOM^b^	1.406 (0.859–2.300)	1.416 (0.863–2.325)	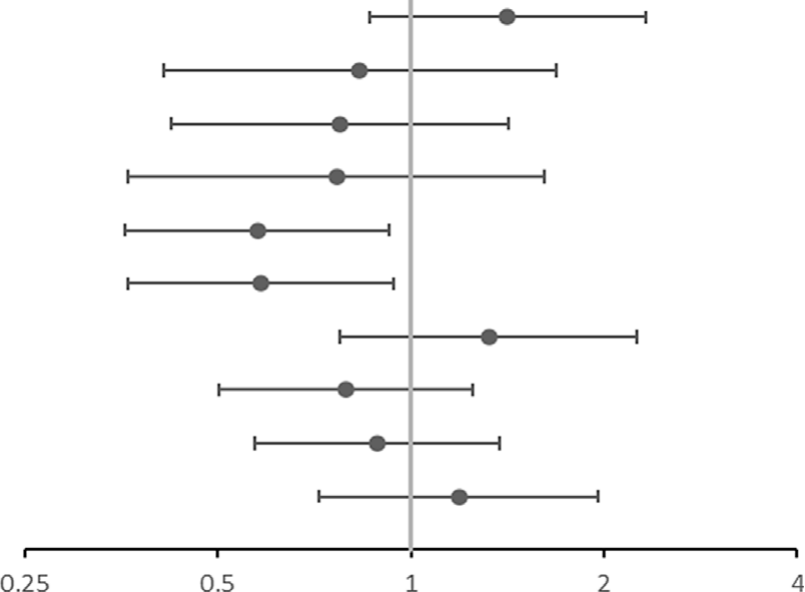 OR corr (95% CI)
rs2381416 REC^c^	0.858 (0.427–1.724)	0.833 (0.413–1.682)	
rs928413 REC^c^	0.798 (0.438–1.454)	0.775 (0.424–1.418)	
rs992969 REC^c^	0.780 (0.371–1.641)	0.766 (0.363–1.616)	
rs10208293 DOM^b^	**0.544 (0.341–0.868)**	**0.577 (0.359–0.927)**	
rs13424006 DOM^b^	**0.552 (0.345–0.882)**	**0.584 (0.362–0.941)**	
rs1420101 REC^c^	1.321 (0.777–2.245)	1.323 (0.776–2.254)	
rs4142132 REC^c^	0.773 (0.491–1.217)	0.792 (0.502–1.251)	
rs9290936 DOM^b^	0.900 (0.581–1.394)	0.887 (0.572–1.378)	
rs10513854 DOM^b^	1.165 (0.718–1.892)	1.188 (0.719–1.962)	

a OR, corr: the *p* value after adjusting age and gender as covariates; OR: Odds ratio (reference group designated with an OR, of 1.0). *p*-values < 0.05 are in bold.

b DOM, means AA vs (Aa + aa).

c REC, means (AA+ Aa) vs aa; “A” is the major allele and “a” is the minor allele.

### Association of IL33-IL1RL1-IL1RAP Complex Snps With Inflammation-Type in Asthmatic Children

Asthmatic children were divided into FeNO increased group (FeNO ≥25 ppb) and FeNO normal group (FeNO<25 ppb). The allele and genotype frequencies of the 10 SNPs in these two groups is presented in Supplementary eTable S1 in the Supplement. Table 5 shows the association between genotypes and increased risk of FeNO in childhood asthma. Children with a CT or TT genotype of rs4742170 in the IL33 gene had a higher risk of FeNO elevation (corrected OR = 2.629, 95%CI: 1.073–6.445, *p* = 0.035). Children with a GA or AA genotype of rs10208293 in IL1RL1 had a lower risk of increased FeNO (corrected OR = 0.271, 95%CI: 0.118–0.621, *p* = 0.002), and both TC and CC genotypes of rs13424006 had a lower risk of increased FeNO in children with asthma (corrected OR = 0.286, 95%CI: 0.125–0.652, *p* = 0.003). However, when asthmatic children were divided into a PBEC increased group (PBEC >300 /μL) and a PBEC normal group (PBEC ≤300 /μL), there was no statistically significant difference in allele or genotype frequencies of these groups (Supplementary eTable S2 in Supplement).

**TABLE 5:** Association of gene SNPs with risk of high FeNO at baseline.

SNP	OR (95%CI)	Adjusted OR (95% CI)^a^	
rs4742170 DOM^b^	**2.583(1.063–6.277)**	**2.629(1.073–6.445)**	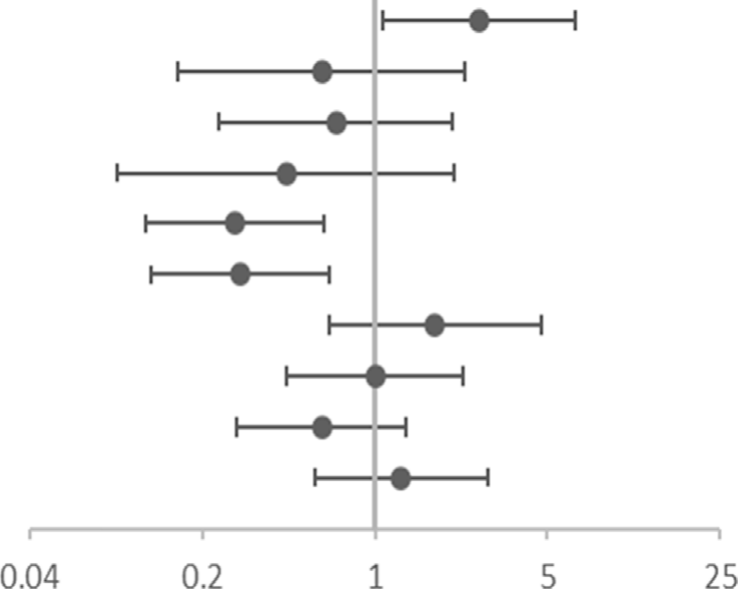 OR corr (95% CI)
rs2381416 REC^c^	0.545 (0.145–2.052)	0.609 (0.159–2.327)	
rs928413 REC^c^	0.618 (0.212–1.797)	0.695 (0.235–2.059)	
rs992969 REC^c^	0.404 (0.085–1.920)	0.439 (0.091–2.109)	
rs10208293 DOM^b^	**0.304(0.136–0.680)**	**0.271(0.118–0.621)**	
rs13424006 DOM^b^	**0.317(0.142–0.708)**	**0.286(0.125–0.652)**	
rs1420101 REC^c^	1.813 (0.681–4.827)	1.756 (0.654–4.717)	
rs4142132 REC^c^	1.093 (0.487–2.452)	1.000 (0.439–2.275)	
rs9290936 DOM^b^	0.604 (0.276–1.326)	0.608 (0.275–1.341)	
rs10513854 DOM^b^	1.156 (0.526–2.540)	1.281 (0.573–2.865)	

a OR, corr: the *p* value after adjusting age and gender as covariates; OR: Odds ratio (reference group designated with an OR, of 1.0). *p*-values < 0.05 are in bold.

b DOM, means AA vs (Aa + aa).

c REC, means (AA+ Aa) vs aa; “A” is the major allele and “a” is the minor allele.

### Association of IL33-IL1RL1-IL1RAP Complex SNPs With the Efficacy of ICS

The efficacy of ICS was evaluated by comparing indicators, including PBEC, FeNO, FEV1/FVC, MMEF/pre, and PD20, before and after treatment with ICS for 3 months.

The risk of no improvement in FeNO in children with CT and TT genotypes of rs4742170 in the IL33 gene, was significantly higher than those children with CC genotypes (corrected OR = 6.510, 95%CI: 1.540–27.527, *p* = 0.011), suggesting that eosinophilic airway inflammation in children with a T allele at rs4742170 would respond poorly to ICS therapy (Table 6). The risk of no improvement in PBEC after treatment for children with a C allele at rs1420101 in IL1RL1 was lower than the other two genotypes (OR = 0.231, 95%CI: 0.059–0.902, *p* = 0.035), suggesting that the TT phenotype of rs1420101 indicated a higher risk of poor response to ICS. Children with a G allele at rs4142132 had a lower risk of no improvement in PBEC after treatment (OR = 0.260, 95%CI: 0.076–0.891, *p* = 0.032), suggesting that children with an AA genotype of rs4142132 had a higher risk of no improvement in PBEC after treatment (Table 7).

**TABLE 6:** Association of gene SNPs with risk of no response to ICS in terms of FeNO in asthmatic children after treatment with ICS for 3 months.

SNP	OR (95%CI)	Adjusted OR (95% CI)^a^	
rs4742170 DOM^b^	6.771 (1.677–27.338)	**6.510(1.540–27.527)**	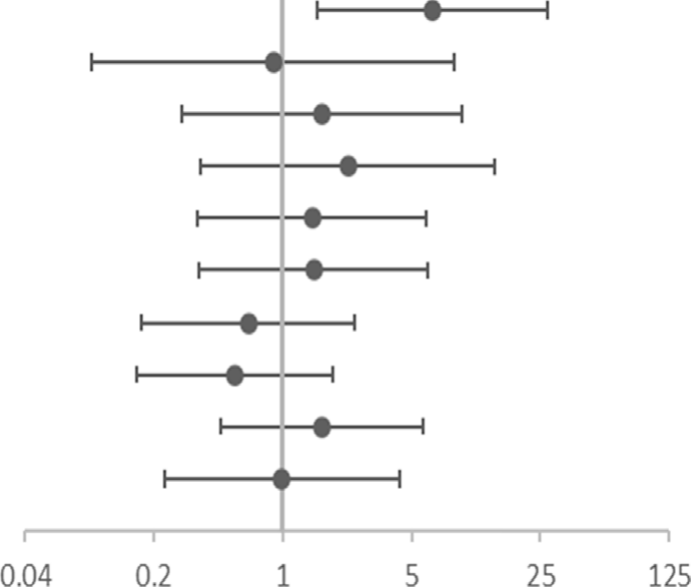 OR corr (95% CI)
rs2381416 REC^c^	1.182 (0.126–11.102)	0.889 (0.092–8.570)	
rs928413 REC^c^	1.909 (0.341–10.701)	1.634 (0.284–9.400)	
rs992969 REC^c^	3.045 (0.497–18.669)	2.253 (0.360–14.109)	
rs10208293 DOM^b^	1.132 (0.280–4.575)	1.437 (0.344–5.997)	
rs13424006 DOM^b^	1.154 (0.285–4.666)	1.472 (0.353–6.144)	
rs1420101 REC^c^	0.614 (0.166–2.272)	0.647 (0.170–2.456)	
rs4142132 REC^c^	0.467 (0.140–1.561)	0.549 (0.160–1.879)	
rs9290936 DOM^b^	1.594 (0.465–5.459)	1.636 (0.464–5.770)	
rs10513854 DOM^b^	1.154 (0.285–4.666)	0.989 (0.229–4.276)	

a OR, corr: the *p* value after adjusting age and gender as covariates; OR: Odds ratio (reference group designated with an OR, of 1.0). *p*-values < 0.05 are in bold.

b DOM, means AA vs (Aa + aa).

c REC, means (AA+ Aa) vs aa; “A” is the major allele and “a” is the minor allele.

**TABLE 7:** Association of gene SNPs with risk of no response to ICS in terms of PBEC in asthmatic children after treatment with ICS for 3 months.

SNP	OR (95%CI)	Adjusted OR (95% CI)^c^	
rs4742170 DOM^a^	1.784 (0.420–7.574)	1.634 (0.373–7.154)	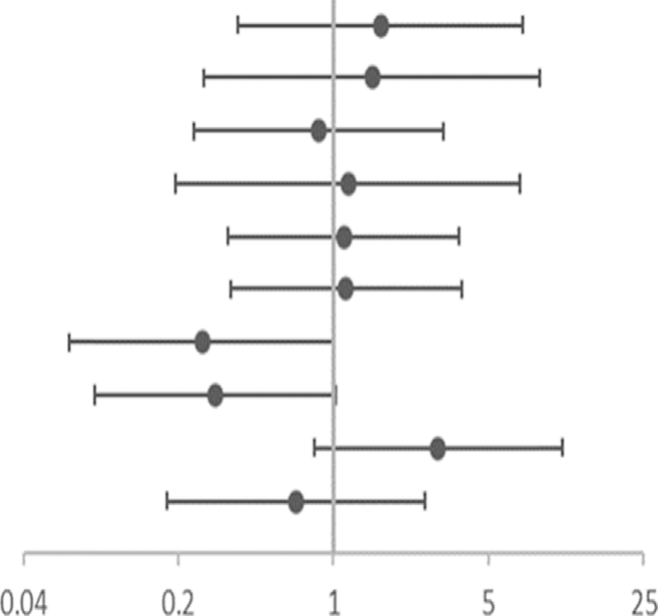 OR corr (95% CI)
rs2381416 REC^b^	1.806 (0.325–10.039)	1.490 (0.262–8.454)	
rs928413 REC^b^	0.947 (0.268–3.351)	0.854 (0.234–3.118)	
rs992969 REC^b^	1.459 (0.249–8.554)	1.160 (0.193–6.959)	
rs10208293 DOM^a^	1.273 (0.403–4.021)	1.109 (0.335–3.673)	
rs13424006 DOM^a^	1.312 (0.416–4.141)	1.137 (0.343–3.766)	
rs1420101 REC^b^	**0.231(0.059**–**0.902)**	0.255 (0.064–1.011)	
rs4142132 REC^b^	**0.260(0.076**–**0.891)**	0.295 (0.084–1.028)	
rs9290936 DOM^a^	3.241 (0.940–11.168)	2.956 (0.817–10.700)	
rs10513854 DOM^a^	0.504 (0.140–1.818)	0.676 (0.177–2.588)	

a DOM, means AA vs (Aa + aa).

b REC, means (AA+ Aa) vs aa; “A” is the major allele and “a” is the minor allele.

c OR, corr: the *p* value after adjusting age and gender as covariates; OR: Odds ratio (reference group designated with an OR, of 1.0). *p*-values < 0.05 are in bold.

The analysis of 10 SNPs and lung function indicators showed that the risk of no improvement in PD20 was significantly higher in children with CT and TT genotypes of rs4742170 in IL33 after treatment (OR = 8.679, 95%CI: 1.029–73.215, *p* = 0.047), suggesting that asthmatic children with a T allele at rs4742170 had a significantly increased risk of poor response to ICS treatment for airway hyperresponsiveness (Table 8). However, similar associations were not found for FEV1/FVC or MMEF/pre.

**TABLE 8:** Association of the IL33 rs4742170 genotype with no improvement in PD20 after ICS treatment for 3 months.

Genotype	OR	95%CI	*p*-value
CC vs (CT + TT)	8.679	(1.029, 73.215)	0.047
(CC + CT) vs TT	4.183	(1.422, 12.304)	0.009

*p*-values < 0.05 are in bold.

## Discussion

As is well known, genetic factors play an important role in susceptibility to asthma. However, studies that focus on the association between genetic variations and asthma susceptibility in Chinese children are still limited. To the best of our knowledge, this is the first study that confirmed that SNPs of IL33/IL1RL1/IL1RAP are associated with Han Chinese childhood asthma. This study provides evidence for the association of asthma susceptibility, eosinophilic airway inflammation, and efficacy of ICS with distinct IL33/IL1RL1 ligand-receptor complex polymorphisms.

SNPs in IL33 previously associated with asthma susceptibility in European populations were not significantly associated with asthma susceptibility in Han children in our study. But we found that children with a T allele at rs4742170 in IL33 had a greater risk of increased levels of the eosinophilic airway inflammation marker FeNO (OR = 2.583, 95%CI: 1.063–6.277, *p* = 0.036), and a worse response to ICS (no improvement in FeNO after ICS treatment: OR = 6.771, 95%CI: 1.677–27.338, *p* = 0.007, no improvement in PD20 after ICS treatment: OR = 4.183, 95%CI: 1.422–12.304, *p* = 0.009). rs4742170 is located on the second intron of the IL33 gene, 27 KB distant from the IL33 promoter (Rosenbloom et al., 2013). The glucocorticoid receptor (GR) is expressed in lung epithelial cells. GR is activated by glucocorticoids and transferred to the nucleus to regulate the expression level of genes involved in stress and steroid responses in inflammation, by binding to nuclear glucocorticoid response elements (Surjit et al., 2011; Gorbacheva et al., 2018). The therapeutic effect of ICS is mainly to inhibit the expression of inflammatory genes through various DNA-binding mechanisms. At present, the specific GR-DNA of GR target genes is unclear. The five prime ends, the first intron and the second intron regions of the IL33 gene have some typical characteristics of regulatory elements (Gorbacheva et al., 2018).

Once the C allele at the rs4742170 site in lung cancer cells mutated to become a T allele, it was observed that the GR binding site was destroyed and the binding force between the GR and the IL33 gene was reduced (Gorbacheva et al., 2018). The activity of the IL33 promoter was enhanced, resulting in increased expression of IL33. The variation of rs4742170 may explain some of the pathophysiological mechanisms of asthma with ICS non-response.

IL1RL1 encodes three protein subtypes:1) IL1RL1-a (also known as soluble ST2), which can be detected in serum; 2) Transmembrane receptor protein IL1RL1-b (S2TL); and 3) IL1RL1-c (ST2V) (Takatori et al., 2018). IL1RL1-a, IL1RL1-b, and IL1RL1-c are all expressed in the lung. IL33 binds with IL1RL1-b, which is widely expressed on the membrane of type 2 immune cells, and then combines with IL1RAP to form a ternary complex, which activates immune cells to release type 2 cytokines such as IL4, IL5, and IL13 to promote airway inflammation. IL1RL1-a is considered to be a decoy receptor that blocks the IL33-IL1RL1 pathway by binding free IL33 and thus downregulates its regulatory ability on immune cells (Hayakawa et al., 2007).

The concentration of IL1RL1-a is greatly influenced by genetic factors. The heritability of IL1RL1-a was found to be 0.45, which means that 45% of the clinically unexplained differences in IL1RL1-a levels are due to genetic factors. In addition, no correlation was found between environmental factors and IL1RL1-a levels (*p* = 0.25) (Ho et al., 2013). The GWAS study reported in 2018 included some loci of the IL1RL1 gene region in the list of asthma highly related SNPs in Europeans (El-Husseini et al., 2020).

rs10208293 and rs13424006 are located in the 10th intron of IL1RL1. Two European birth cohorts and one paternity cohort study showed that these two sites were associated with late-onset wheezing in children (OR = 0.74, 95%CI: 0.62–0.87, *p* < 0.01) (Savenije et al., 2014). In our study, these two sites were associated with susceptibility to childhood asthma and eosinophilic airway inflammation in Han Chinese children. We speculate that these two SNPs may be involved in the regulation of IL1RL1-a.

rs1420101, located in the fifth intron of IL1RL1, has been widely validated in multiple GWAS studies (Demenais et al., 2018; Dahlin et al., 2019). A Dutch cohort and three European birth cohorts showed that SNPs in the IL1RL1 gene region were significantly correlated with gene methylation, and effectively regulated serum IL1RL1-a levels (Dijk et al., 2018). Studies have found that the T allele of rs1420101 in Europeans is not only a risk allele for asthma, but is also associated with higher eosinophil counts and higher IgE levels (Gudbjartsson et al., 2009; Grotenboer et al., 2013). Further studies have found that the T allele at rs1420101 was associated with lower levels of DNA methylation, low expression of IL1RL1 mRNA in airway epithelial cells and lung tissue, and reduced levels of IL1RL1-a in serum and alveolar lavage fluid (Akhabir and Sandford, 2010; Gordon et al., 2016; Dijk et al., 2018). The decrease inIL1RL1-a levels resulted in the upregulation of IL33 and increased IL1RL1-b binding force and the enhancement of type 2 inflammatory response. In our study, rs1420101 was not significantly correlated with asthma susceptibility or PBEC in Han children in the Hunan region. However, it was observed that children with asthma who had the TT genotype of rs1420101 or AA genotype ofrs4142132 in the first intron of IL1RL1, responded better to ICS treatment in PBEC. Therefore, we speculate that ICS can effectively control asthma with rs1420101 and rs4142132 mutations, through inhibiting the expression of upstream IL33.

There are few studies examining SNPs in the IL1RAP gene. IL1RAP is an essential co-receptor in the interleukin-1 family (Fields et al., 2019). Recent studies have found that, besides the membrane receptor, L1RAcP encodes a kind of soluble protein that inhibits the inflammatory response through enhancing the binding of Il1RL1-a with IL33 (Palmer et al., 2008, 33). rs9290936 in the first intron of the IL1RAP gene was associated with persistent wheezing in children in Dutch and UK populations (OR = 0.72, 95%CI: 0.61–0.85, *p* < 0.01), and rs10513854 in the second intron was associated with intermediate-onset wheezing (OR = 0.67, 95%CI: 0.55-0.83, *p* < 0.01) (Savenije et al., 2014). However, we did not find any association between these two SNPs and childhood asthma in Han children.

## Conclusion

In conclusion, we found significant associations between IL1RL1 rs10208293 and rs13424006 polymorphisms and the risk of childhood asthma. In addition, we found that they are associated with type 2 airway inflammation, which is like rs4742170 in IL33. When it comes to the efficacy of ICS, rs4742170 in IL33, rs1420101, and rs4142132 in IL1RL1 polymorphisms were significantly associated in our study. No association between asthma and either SNP in IL1RAP was observed. Our results indicated that rs10208293 and rs13424006 could be asthma markers in children, while rs4742170, rs1420101, and rs4142132 might be used to predict the ICS response in childhood asthma.

## Data Availability

The original contributions presented in the study are included in the article/Supplementary Material, further inquiries can be directed to the corresponding author.

## Abbreviations

FeNO, fractional exhaled nitric oxide; FEV1, forced expiratory volume in 1 s; FVC, forced vital capacity; GR, glucocorticoid receptor; ICS, inhaled corticosteroid; IL1RL1, interleukin-1 receptor-like 1 protein; IL1RAP, interleukin-1 receptor accessory protein; IL33, interleukin 33; MMEF, maximal mid-expiratory flow; PBEC, peripheral blood eosinophil count; PD20, provocative dose of methacholine causing a 20% drop in FEV1; SNPs, single nucleotide polymorphisms.
