# Supplementary material for: Association of IL33, IL1RL1, IL1RAP Polymorphisms and Asthma in Chinese Han Children
Source: Front Cell Dev Biol. 2021 Dec 15;9:759542. doi: 10.3389/fcell.2021.759542 (PMC8714920; doi:10.3389/fcell.2021.759542)
Supplement: Supplementary file 1 [file Table1.docx]

eTable 1. The allele and genotype frequency of 10 SNPs in asthmatic children with normal FeNO and high FeNO on the baseline.

| Gene | SNP | Genotype/ Allele | Normal ^a^FeNO  (N=49) | |  | Increased ^a^FeNO（N=105） | | *p*-value | ^b^DOM  model *p*-value | ^c^REC  model *p*-value |
| --- | --- | --- | --- | --- | --- | --- | --- | --- | --- | --- |
|  |  |  | N | （%） |  | N | （%） |  |  |  |
| IL33 | rs4742170 | Total | 48 | (100.00) |  | 105 | (100.00) | 0.073 | **0.032** | 0.843 |
|  |  | CC | 12 | (25.00) |  | 12 | (11.43) |  |  |  |
|  |  | TC | 22 | (45.83) |  | 64 | (60.95) |  |  |  |
|  |  | TT | 14 | (29.17) |  | 29 | (27.62) |  |  |  |
|  |  | C | 46 | (47.92) |  | 88 | (41.90) | 0.325 |  |  |
|  |  | T | 50 | (52.08) |  | 122 | (58.10) |  |  |  |
|  | rs2381416 | Total | 49 | (100.00) |  | 103 | (100.00) | 0.550 | / | 0.382 |
|  |  | AA | 46 | (93.88) |  | 92 | (89.32) |  |  |  |
|  |  | CA | 3 | (6.12) |  | 11 | (10.68) |  |  |  |
|  |  | CC | 0 | (0.00) |  | 0 | (0.00) |  |  |  |
|  |  | A | 95 | (96.94) |  | 195 | (94.66) | 0.560 |  |  |
|  |  | C | 3 | (3.06) |  | 11 | (5.34) |  |  |  |
|  | rs928413 | Total | 49 | (100.00) |  | 103 | (100.00) | 0.431 | 0.536 | 0.397 |
|  |  | AA | 44 | (89.80) |  | 87 | (84.47) |  |  |  |
|  |  | GA | 4 | (8.16) |  | 15 | (14.56) |  |  |  |
|  |  | GG | 1 | (2.04) |  | 1 | (0.97) |  |  |  |
|  |  | A | 92 | (93.88) |  | 189 | (91.75) | 0.512 |  |  |
|  |  | G | 6 | (6.12) |  | 17 | (8.25) |  |  |  |
|  | rs992969 | Total | 49 | (100.00) |  | 105 | (100.00) | 0.082 | 0.314 | 0.342 |
|  |  | AA | 1 | (2.04) |  | 0 | (0.00) |  |  |  |
|  |  | AG | 1 | (2.04) |  | 10 | (9.52) |  |  |  |
|  |  | GG | 47 | (95.92) |  | 95 | (90.48) |  |  |  |
|  |  | A | 3 | (3.06) |  | 10 | (4.76) | 0.762 |  |  |
|  |  | G | 95 | (96.94) |  | 200 | (95.24) |  |  |  |
| IL1RL1 | rs10208293 | Total | 48 | (100.00) |  | 105 | (100.00) | **0.005** | **0.002** | 0.134 |
|  |  | AA | 1 | (2.08) |  | 0 | (0.00) |  |  |  |
|  |  | AG | 16 | (33.33) |  | 15 | (14.29) |  |  |  |
|  |  | GG | 31 | (64.58) |  | 90 | (85.71) |  |  |  |
|  |  | A | 18 | (18.75) |  | 15 | (7.14) | **0.002** |  |  |
|  |  | G | 78 | (81.25) |  | 195 | (92.86) |  |  |  |
|  | rs13424006 | Total | 49 | (100.00) |  | 104 | (100.00) | **0.006** | **0.003** | 0.316 |
|  |  | CC | 1 | (2.04) |  | 0 | (0.00) |  |  |  |
|  |  | CT | 16 | (32.65) |  | 15 | (14.42) |  |  |  |
|  |  | TT | 32 | (65.31) |  | 89 | (85.58) |  |  |  |
|  |  | C | 18 | (18.37) |  | 15 | (7.21) | **0.003** |  |  |
|  |  | T | 80 | (81.63) |  | 193 | (92.79) |  |  |  |
|  | rs1420101 | Total | 49 | (100.00) |  | 104 | (100.00) | 0.326 | 0.188 | 0.249 |
|  |  | CC | 19 | (38.78) |  | 30 | (28.85) |  |  |  |
|  |  | TC | 24 | (48.98) |  | 53 | (50.96) |  |  |  |
|  |  | TT | 6 | (12.24) |  | 21 | (20.19) |  |  |  |
|  |  | C | 62 | (63.27) |  | 113 | (54.33) | 0.140 |  |  |
|  |  | T | 36 | (36.73) |  | 95 | (45.67) |  |  |  |
|  | rs4142132 | Total | 49 | (100.00) |  | 104 | (100.00) | 0.764 | 0.599 | 0.88 |
|  |  | AA | 11 | (22.45) |  | 25 | (24.04) |  |  |  |
|  |  | GA | 27 | (55.10) |  | 51 | (49.04) |  |  |  |
|  |  | GG | 11 | (22.45) |  | 28 | (26.92) |  |  |  |
|  |  | A | 49 | (50.00) |  | 101 | (48.56) | 0.814 |  |  |
|  |  | G | 49 | (50.00) |  | 107 | (51.44) |  |  |  |
| IL1RAP | rs9290936 | Total | 49 | (100.00) |  | 105 | (100.00) | 0.124 | 0.233 | 0.054 |
|  |  | GG | 11 | (22.45) |  | 34 | (32.38) |  |  |  |
|  |  | GT | 28 | (57.14) |  | 61 | (58.10) |  |  |  |
|  |  | TT | 10 | (20.41) |  | 10 | (9.52) |  |  |  |
|  |  | G | 50 | (51.02) |  | 129 | (61.43) | 0.085 |  |  |
|  |  | T | 48 | (48.98) |  | 81 | (38.57) |  |  |  |
|  | rs10513854 | Total | 49 | (100.00) |  | 99 | (100.00) | 0.689 | 0.572 | 0.551 |
|  |  | TT | 0 | (0.00) |  | 3 | (3.03) |  |  |  |
|  |  | TC | 12 | (24.49) |  | 24 | (24.24) |  |  |  |
|  |  | CC | 37 | (75.51) |  | 72 | (72.73) |  |  |  |
|  |  | T | 12 | (12.24) |  | 30 | (15.15) | 0.500 |  |  |
|  |  | C | 86 | (87.76) |  | 168 | (84.85) |  |  |  |

If the total number of normal FeNO is not 49, or increased FeNO is not 105, it is due to missing data. *p*-values< 0.05 are in bold. ^a^FeNO, fractional exhaled nitric oxide; ^b^DOM means AA vs (Aa + aa); ^c^REC means (AA + Aa) vs aa; “A” is the major allele, and “a” is the minor allele.

eTable 2. The allele and genotype frequency of 10 SNPs in asthmatic children with normal PBEC and high PBEC on the baseline.

| Gene | SNP | Genotype/ Allele | Normal ^a^PBEC  (N=113) | |  | Increased ^a^PBEC（N=105） | | *p*-value | ^b^DOM  model *p*-value | ^c^REC  model *p*-value |
| --- | --- | --- | --- | --- | --- | --- | --- | --- | --- | --- |
|  |  |  | N | （%） |  | N | （%） |  |  |  |
| IL33 | rs4742170 | Total | 112 | (100.00) |  | 104 | (100.00) | 0.910 | 0.665 | 0.930 |
|  |  | CC | 19 | (16.96) |  | 20 | (19.23) |  |  |  |
|  |  | TC | 59 | (52.68) |  | 53 | (50.96) |  |  |  |
|  |  | TT | 34 | (30.36) |  | 31 | (29.81) |  |  |  |
|  |  | C | 97 | (43.30) |  | 93 | (44.71) | 0.768 |  |  |
|  |  | T | 127 | (56.70) |  | 115 | (55.29) |  |  |  |
|  | rs2381416 | Total | 110 | (100.00) |  | 104 | (100.00) | 0.727 | 0.486 | 0.925 |
|  |  | AA | 99 | (90.00) |  | 94 | (90.38) |  |  |  |
|  |  | CA | 11 | (10.00) |  | 9 | (8.65) |  |  |  |
|  |  | CC | 0 | (0.00) |  | 1 | (0.96) |  |  |  |
|  |  | A | 209 | (95.00) |  | 197 | (94.71) | 0.893 |  |  |
|  |  | C | 11 | (5.00) |  | 11 | (5.29) |  |  |  |
|  | rs928413 | Total | 112 | (100.00) |  | 103 | (100.00) | 0.921 | 1.000 | 0.741 |
|  |  | AA | 95 | (84.82) |  | 89 | (86.41) |  |  |  |
|  |  | GA | 16 | (14.29) |  | 13 | (12.62) |  |  |  |
|  |  | GG | 1 | (0.89) |  | 1 | (0.97) |  |  |  |
|  |  | A | 206 | (91.96) |  | 191 | (92.72) | 0.769 |  |  |
|  |  | G | 18 | (8.04) |  | 15 | (7.28) |  |  |  |
|  | rs992969 | Total | 112 | (100.00) |  | 105 | (100.00) | 0.901 | 1.000 | 0.698 |
|  |  | AA | 1 | (0.89) |  | 1 | (0.95) |  |  |  |
|  |  | AG | 8 | (7.14) |  | 9 | (8.57) |  |  |  |
|  |  | GG | 103 | (91.96) |  | 95 | (90.48) |  |  |  |
|  |  | A | 10 | (4.46) |  | 11 | (5.24) | 0.707 |  |  |
|  |  | G | 214 | (95.54) |  | 199 | (94.76) |  |  |  |
| IL1RL1 | rs10208293 | Total | 112 | (100.00) |  | 104 | (100.00) | 0.054 | 0.068 | 1.000 |
|  |  | AA | 1 | (0.89) |  | 0 | (0.00) |  |  |  |
|  |  | AG | 15 | (13.39) |  | 25 | (24.04) |  |  |  |
|  |  | GG | 96 | (85.71) |  | 79 | (75.96) |  |  |  |
|  |  | A | 17 | (7.59) |  | 25 | (12.02) | 0.120 |  |  |
|  |  | G | 207 | (92.41) |  | 183 | (87.98) |  |  |  |
|  | rs13424006 | Total | 112 | (100.00) |  | 104 | (100.00) | 0.054 | 0.068 | 1.000 |
|  |  | CC | 1 | (0.89) |  | 0 | (0.00) |  |  |  |
|  |  | CT | 15 | (13.39) |  | 25 | (24.04) |  |  |  |
|  |  | TT | 96 | (85.71) |  | 79 | (75.96) |  |  |  |
|  |  | C | 17 | (7.59) |  | 25 | (12.02) | 0.120 |  |  |
|  |  | T | 207 | (92.41) |  | 183 | (87.98) |  |  |  |
|  | rs1420101 | Total | 112 | (100.00) |  | 104 | (100.00) | 0.244 | 0.939 | 0.109 |
|  |  | CC | 35 | (31.25) |  | 33 | (31.73) |  |  |  |
|  |  | TC | 50 | (44.64) |  | 55 | (52.88) |  |  |  |
|  |  | TT | 27 | (24.11) |  | 16 | (15.38) |  |  |  |
|  |  | C | 120 | (53.57) |  | 121 | (58.17) | 0.336 |  |  |
|  |  | T | 104 | (46.43) |  | 87 | (41.83) |  |  |  |
|  | rs4142132 | Total | 111 | (100.00) |  | 104 | (100.00) | 0.560 | 0.970 | 0.315 |
|  |  | AA | 30 | (27.03) |  | 22 | (21.15) |  |  |  |
|  |  | GA | 53 | (47.75) |  | 56 | (53.85) |  |  |  |
|  |  | GG | 28 | (25.23) |  | 26 | (25.00) |  |  |  |
|  |  | A | 113 | (50.90) |  | 100 | (48.08) | 0.558 |  |  |
|  |  | G | 33 | 40.24 |  | 29 | 51.79 |  |  |  |
| IL1RAP | rs9290936 | Total | 112 | (100.00) |  | 105 | (100.00) | 0.418 | 0.413 | 0.457 |
|  |  | GG | 40 | (35.71) |  | 32 | (30.48) |  |  |  |
|  |  | GT | 53 | (47.32) |  | 59 | (56.19) |  |  |  |
|  |  | TT | 19 | (16.96) |  | 14 | (13.33) |  |  |  |
|  |  | G | 133 | (59.38) |  | 123 | (58.57) | 0.865 |  |  |
|  |  | T | 91 | (40.63) |  | 87 | (41.43) |  |  |  |
|  | rs10513854 | Total | 109 | (100.00) |  | 99 | (100.00) | 0.759 | 0.478 | 1.000 |
|  |  | TT | 1 | (0.92) |  | 1 | (1.01) |  |  |  |
|  |  | TC | 29 | (26.61) |  | 22 | (22.22) |  |  |  |
|  |  | CC | 79 | (72.48) |  | 76 | (76.77) |  |  |  |
|  |  | T | 31 | (14.22) |  | 24 | (12.12) | 0.528 |  |  |
|  |  | C | 187 | (85.78) |  | 174 | (87.88) |  |  |  |

If the total number of normal PBEC is not 113 or increased PBEC is not 105, it is due to missing data. *p*-values< 0.05 are in bold. ^a^PBEC, Peripheral blood eosinophil count; ^b^DOM means AA vs (Aa + aa); ^c^REC means (AA + Aa) vs aa; “A” is the major allele, and “a” is the minor allele.
